# Simulation of SAW Sensors with Various Distributed Mass Loadings Using Two-Dimensional Coupling-of-Modes Theory

**DOI:** 10.3390/s20247260

**Published:** 2020-12-18

**Authors:** Ran You, Jiuling Liu, Minghua Liu, Zhiyuan Chen, Shitang He

**Affiliations:** 1 Institute of Acoustics, Chinese Academy of Sciences, Beijing 100190, China; youran@mail.ioa.ac.cn (R.Y.); liuminghua@mail.ioa.ac.cn (M.L.); chenzhiyuan@mail.ioa.ac.cn (Z.C.); heshitang@mail.ioa.ac.cn (S.H.); 2 School of Electronic, Electrical and Communication Engineering, University of Chinese Academy of Sciences, Beijing 100049, China

**Keywords:** SAW sensors, non-uniform load, 2-D COM theory, finite element method (FEM), transverse modes

## Abstract

In order to accurately investigate the disturbance of complex distributed mass loading on surface acoustic wave (SAW) propagation characteristics, two-dimensional coupling-of-modes (2-D COM) theory and finite element method (FEM) were used to simulate the responses of SAW sensors. By using the PDE mode of FEM software, four SAW resonators with the loads in different distribution patterns were modeled. Also, we fabricated and measured a series of SAW resonators accordingly. The results showed that the 2-D COM theory combined with the finite element method was able to simulate the transverse modes of the device and the disturbance of the mass loading on the transverse mode effectively, making the simulation more accurate.

## 1. Introduction

Coupling-of-modes (COM) theory is an effective method for the simulation of surface acoustic wave (SAW) devices [1], which is widely used in the design and optimization of SAW filters. In order to calculate the influence of waveguide effect on the response of SAW filter, Haus et al. proposed the two-dimensional coupling-of-modes (2-D COM) theory [2,3], and then the two-dimensional P-matrix model was developed by Bergmann et al. [4] Although these methods have the ability to calculate the transverse modes of the SAW, they are difficult to be used in the design and optimization of SAW devices effectively due to the complexity of modeling and calculation. In order to improve the efficiency of simulation, Hirota et al. solved the 2-D COM equation by using the finite element method (FEM) [5]. Based on this work, a new form of 2-D COM equations under biquadratic approximation was developed by Xiao et al. They used the finite element method to extract anisotropic parameters, which further improved the accuracy of the two-dimensional simulation [6].

**Figure 1:**
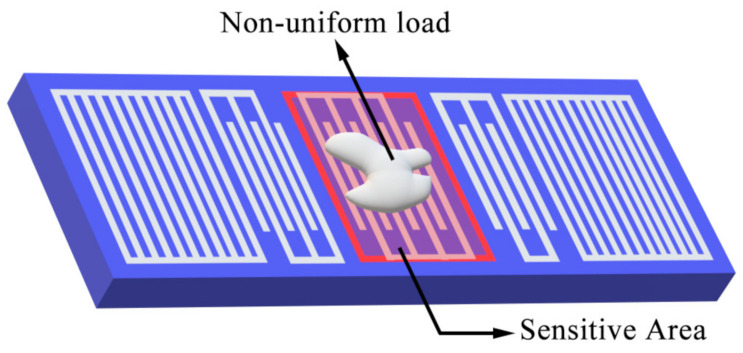
SAW sensors with non-uniform load.

In recent years, the SAW devices have been widely used in the field of biochemical detection [7,8,9,10,11,12]. In many cases, the target analytes adhered to the sensor’s surface usually presents complex and non-uniformly distributions, as shown in Figure 1. Therefore, to investigate the disturbance of complex distributed mass loading on the SAW propagation characteristics is necessary, which put forward higher requirements for the simulation technology. In our previous work, we used a two-dimensional segmentation method (2-D SM) combined with 1-D COM theory to calculate the sensor response under various non-uniformly distributed loads [13]. However, the 2-D SM is an approximate method which discretizes the sensitive area of the sensor, and the 1-D COM theory is unable to simulate the transverse mode of the SAW, so the accuracy of the 2-D SM still needs to be improved. What’s more, the complexity of the two-dimensional segmentation design and the P-matrix cascading operation limit the application of the 2-D SM in different SAW devices.

In order to investigate the disturbance of complex distributed mass loading on the SAW propagation characteristics more accurately and improve the efficiency of simulation, we applied the 2-D COM theory to the simulation of a series of Rayleigh type SAW (R-SAW) sensors in this paper, and used the finite element method to solve the 2-D COM equations. Firstly, the structure model of SAW sensors with four patterns of the mass loading’s distribution were established by using the finite element simulation software (COMSOL Multiphysics); Next, the 2-D COM equation was introduced into the FEM model in the form of a generalized partial differential equation and solved. Finally, the displacement distributions of the main modes of the sensor under four load distribution patterns were drawn according to the solution results. At the same time, we calculated the insertion loss (S21) parameters of the device, and obtained the sensors’ responses under four patterns of the load’s distribution.

To verify the accuracy of simulation results, we fabricated a series of SAW sensor chips with the loads in four distribution patterns, and measured their frequency responses. The experimental results confirm the advantage of using the 2-D COM equations to simulate the disturbance of complex distributed mass loading on SAW propagation characteristics.

## 2. Methods

### 2.1. Two-Dimensional Coupling-of-Modes Equations

**Figure 2:**
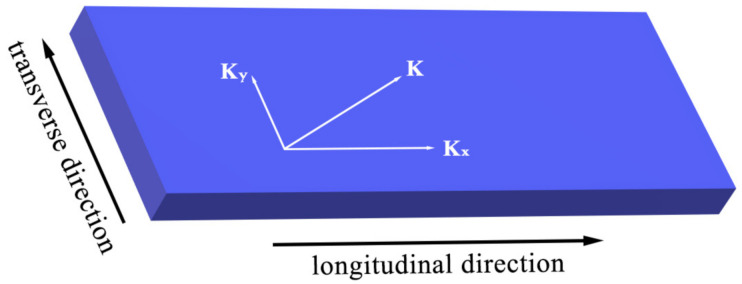
Decomposition of the wave vector **K**.

The 1-D COM theory was based on the plane-wave assumption that ignored the diffraction phenomenon in the propagation of the SAW. Thus, it can’t simulate the transverse mode of the device. In the 2-D COM theory, the surface acoustic wave is no longer approximated to a plane wave whose wave vector **K** is decomposed into two orthogonal components **K**_x_ and **K**_y_, corresponding to the longitudinal and transverse components of the device, respectively, as shown in Figure 2.

According to the slowness curve of the SAW on piezoelectric substrate, the relationship between wave vector component **K**_x_ and **K**_y_ will be established approximately. The parabola approximate form [14] was shown in Equation (1). And the biquadratic approximate form [6] was shown in Equation (2).

(1) $$k_{x}\approx k_{0}-\frac{\gamma }{2}\frac{{\left(k_{y}\right)}^{2}}{k_{0}}$$

(2) $$k_{x}\approx k_{0}+\frac{1\gamma_{1}}{2k_{0}}k_{y}{}^{2}+\frac{1\gamma_{2}}{24k_{0}{}^{3}}k_{y}{}^{4}$$

Here, γ, $\gamma_{1}$, $\gamma_{2}$ are the parameters to characterize the anisotropy of piezoelectric substrate; $k_{0}$ is the wave number according to the electric period of the IDT.

Based on the 1-D COM equation and the slowness relation of the SAW, the 2-D COM equation can be obtained. As shown in Equation (3), the 2-D COM equations in the form of biquadratic approximation is adopted in this paper.

(3) $$\left\{\begin{array}{l} \frac{\partial u_{+}}{\partial x}=-jk_{0}u_{+}+j\frac{\gamma_{1}}{2k_{0}}\frac{\partial^{2}u_{+}}{\partial y^{2}}+j\frac{\gamma_{1}}{24k_{0}{}^{3}}\frac{\partial^{4}u_{+}}{\partial y^{4}}+j\kappa u_{-}+j\alpha V\\ \frac{\partial u_{-}}{\partial x}=jk_{0}u_{-}-j\frac{\gamma_{1}}{2k_{0}}\frac{\partial^{2}u_{-}}{\partial y^{2}}-j\frac{\gamma_{2}}{24k_{0}{}^{3}}\frac{\partial^{4}u_{-}}{\partial y^{4}}-j\kappa^{*}u_{-}-j\alpha^{*}V\\ \frac{\partial^{2}I}{\partial x\partial y}=-2j\alpha^{*}u_{+}-2j\alpha u_{-}+j\omega CV \end{array}\right.$$

Here, $u_{+}$, $u_{-}$ are the particle displacements of waves propagating forward and backward directions, respectively; κ, α, C, V and I represent coupling coefficient, excitation coefficient, static capacitance, input voltage and output current.

### 2.2. Two-Dimensional Finite Element Model of the SAW Sensor Chip

**Figure 3:**
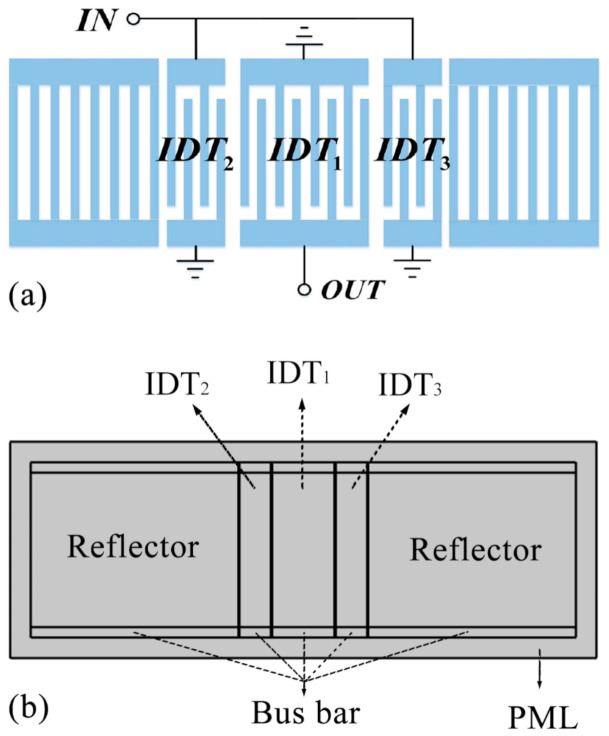
(**a**) The structure of a two-port R-SAW resonator with three IDTs; (**b**) The 2-D model of the SAW resonator built in FEM software.

In this paper, a two-port SAW resonator was selected as the sensing chip and its structure was shown in Figure 3a. The 2-D model of the SAW resonator was built in the finite element simulation software, as shown in Figure 3b. The structural parameters were given in Table 1.

**Figure 4:**
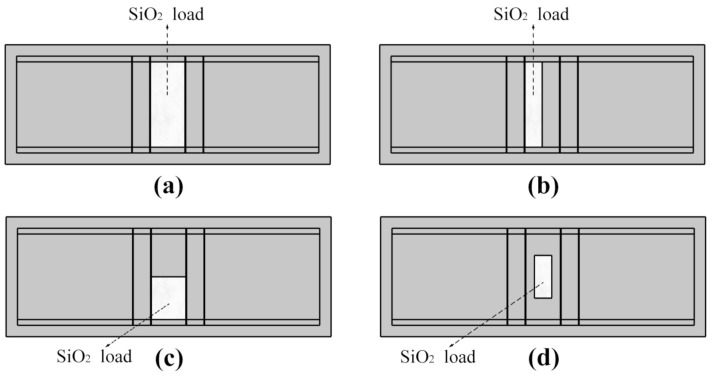
Geometries of SAW resonators with four different load-distribution patterns. (**a**) Load pattern-1; (**b**) Load pattern-2; (**c**) Load pattern-3; (**d**) Load pattern-4.

To investigate the disturbance of complex distributed mass loading on the SAW propagation characteristics, four different load-distribution patterns were designed, as shown in Figure 4. The white parts were the mass loadings.

### 2.3. Two-Dimensional COM Parameters

The 2-D COM parameters are composed of the 1-D COM parameters *v*, *κ*, *α*, *C* and the anisotropic parameters $\gamma_{2}$, $\gamma_{2}$. The 1-D COM parameters can be determined by Equation (4).

(4) $$\left\{\begin{array}{l} v=\frac{(f_{sc+}+f_{sc-})\lambda }{2}\\ \left|\kappa \right|=\frac{2\pi (f_{sc+}-f_{sc-})}{(f_{sc+}+f_{sc-})\lambda }\\ \left|\alpha \right|=\sqrt{\frac{\omega CW\pi }{\lambda^{2}}(\frac{f_{oc+}+f_{oc-}}{f_{sc+}+f_{sc-}}-1)}\\ C=\frac{2W_{e}}{V^{2}W} \end{array}\right.$$

Here, $f_{sc+}$, $f_{sc-}$, $f_{oc+}$, and $f_{oc-}$ denote the up and down boundary frequency of the stop band in periodic shorted grating and open grating; V, W and $W_{e}$ represent the input voltage, the acoustic aperture and the electrostatic energy.

**Figure 5:**
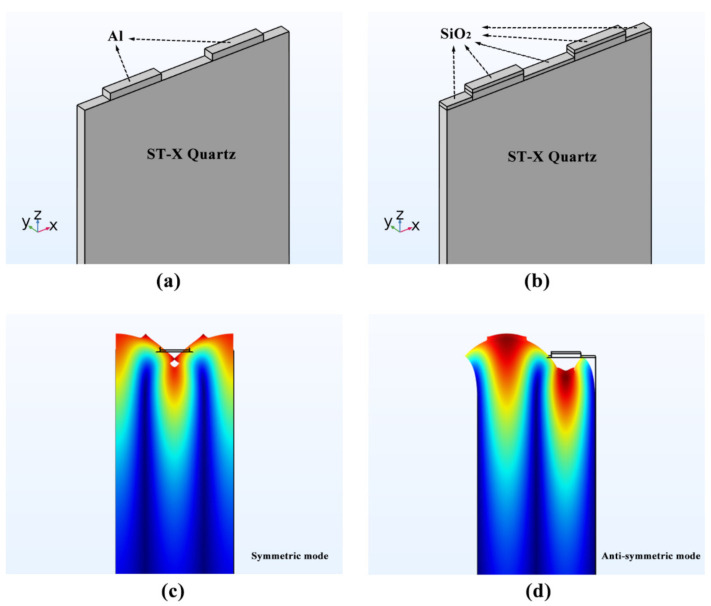
3D FEM model of periodic grating structure and Rayleigh surface acoustic wave (R-SAW) modes: (**a**) periodic gratings without SiO_2_ layer (**b**) periodic gratings with SiO_2_ layer (**c**) symmetric R-SAW mode (**d**) anti-symmetric R-SAW mode.

To determine $f_{sc+}$, $f_{sc-}$, $f_{oc+}$, $f_{oc-}$ and $W_{e}$, a 3D periodic finite element model, including a pair of IDTs, was established as shown in Figure 5. And the substrate material and structural parameters of the model were given in Table 2.

By setting the boundary conditions of constant voltage or constant charge electrode, the periodic grating was controlled to be shorted grating and open grating. The resonant frequencies of symmetric and anti-symmetric Rayleigh surface acoustic wave (R-SAW) modes corresponding to the up and down boundary frequency of the stop band.

By performing static analysis in the FEM software (COMSOL Multiphysics), the electrostatic energy of the periodic element $W_{e}$ can be obtained.

The 1-D COM parameters of IDTs with and without mass loading were calculated by Equation (4), as shown in Table 3.

By calculating the second and fourth derivatives at the zero point of the slowness curve, the anisotropic parameters were obtained [6].

(5) γ1=d2(kxk0)d(kyk0)2ky=0

(6) γ2=d4(kxk0)d(kyk0)4ky=0

To obtain the slowness curves of the SAW, the periodic conditions of the 3D periodic FEM model at the front and back side should be set up to a Floquet-Bloch boundary conditions form as follows:

(7) $$U_{F}=U_{B}\exp(jk_{0}\tan(\theta )y)$$

Here, $U_{F}$ and $U_{B}$ denote the wave amplitudes at front side and back side, and θ is the oblique angle.

By calculating the slowness of the SAW with different oblique angles, the slowness curves were obtained.

**Figure 6:**
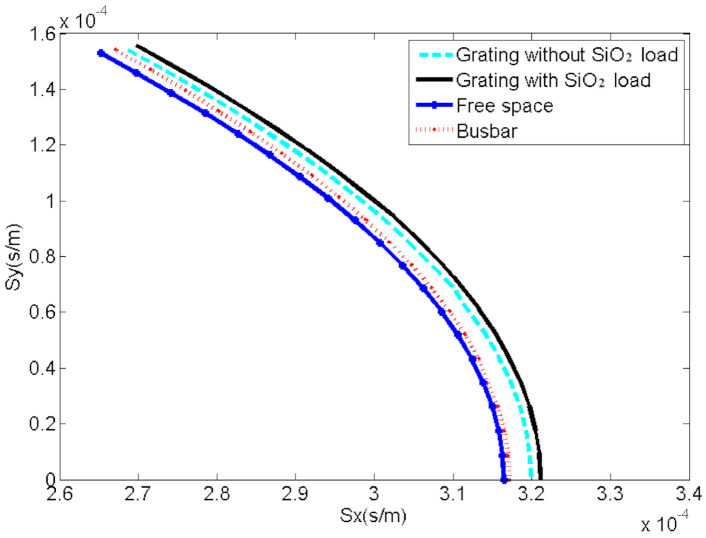
The slowness curves of the SAW in different domains.

The slowness curves of the SAW in different domains on ST-X quartz substrate were shown in Figure 6.

The anisotropic parameters $\gamma_{1}$ and $\gamma_{2}$ of each domain were obtained by Equations (5) and (6), as shown in Table 4.

### 2.4. Generalized PDE Form of 2-D COM Equations

In the PDE mode of FEM software (COMSOL Multiphysics), the 2-D COM equations in the form of biquadratic approximation were established into generalized PDE form [6].

The SAW displacements $U_{+}$ and $U_{-}$ in Equation (3) could be represented as Equation (8):

(8) $$\begin{array}{l} U_{+}=A_{1}\exp(-jk_{0}x)\\ U_{-}=A_{2}\exp(jk_{0}x) \end{array}$$

Then, the 2-D COM equations were rewritten as follows:

(9) $$\left[\begin{array}{l} \nabla \cdot \left[\begin{array}{c} A_{1}\\ -j\frac{\gamma_{2}}{24k_{0}{}^{3}}\frac{\partial A_{3}}{\partial y} \end{array}\right]=-j\Delta A_{1}-j\kappa A_{2}+j\frac{\gamma_{1}}{2k_{0}}A_{3}+\alpha V\\ \nabla \cdot \left[\begin{array}{c} A_{2}\\ -j\frac{\gamma_{2}}{24k_{0}{}^{3}}\frac{\partial A_{4}}{\partial y} \end{array}\right]=-j\Delta A_{2}-j\kappa^{*}A_{1}+j\frac{\gamma_{1}}{2k_{0}}A_{4}+\alpha^{*}V\\ \nabla \cdot \left[\begin{array}{c} 0\\ \frac{\partial A_{1}}{\partial y} \end{array}\right]=A_{3}\\ \nabla \cdot \left[\begin{array}{c} 0\\ \frac{\partial A_{2}}{\partial y} \end{array}\right]=A_{4}\\ \Delta =\frac{\omega }{v}-k_{0} \end{array}\right.$$

Here, ∇ was the divergence operator, Δ represented detuning coefficient.

In the reflector domains, the electric terms were equal to zero due to the electrical shorting of the gratings. In the gaps, PML (perfectly matched layer) and busbar domains, the electric terms and internal reflection terms were equal to zero because there were no internal reflections. What’s more, the PML domain was set to avoid reflections from the boundaries, thus a decay factor $\gamma_{\alpha }(r)$ was introduced into the detuning coefficient of PML domain, as shown in Equation (10).

(10) $$\left[\begin{array}{l} \Delta_{PML}=\frac{\omega }{v}\left[1-j\gamma_{\alpha }(r)\right]-k_{0}\\ \gamma_{\alpha }(r)=\gamma_{0}\frac{r}{d} \end{array}\right.$$

Here, d represented the width of the PML domain, and r was the distance from the boundary. The $\gamma_{0}$ in this paper was set up to 0.5 to achieve sufficient absorption effect.

### 2.5. Admittance Matrix and Insertion Loss

For the two-port SAW resonator, the relationship between the output current *I* and the input voltage *V* was given by Equation (11):

(11) $$\left(\begin{array}{l} I_{1}\\ I_{2} \end{array}\right)=Y\left(\begin{array}{l} V_{1}\\ V_{2} \end{array}\right)=\left(\begin{array}{cc} Y_{11} & Y_{12}\\ Y_{21} & Y_{22} \end{array}\right)\left(\begin{array}{l} V_{1}\\ V_{2} \end{array}\right)$$

Here, *Y* matrix was the admittance matrix of the device.

The current of each port was obtained by Equation (12):

(12) $$I=\iint_{Domain}-2j\alpha^{*}u_{+}-2j\alpha u_{-}+j\omega CV$$

By short-circuiting the IDTs of the two ports respectively and calculating the current of each port, the admittance matrix was obtained.

The principle of the short-circuiting operation to calculate the admittance matrix’s elements was summarized in Table 5.

In this paper, the insertion loss (S_21_) parameter of SAW resonator was chosen as the response signal of the sensor. The relationship between the insertion loss (S_21_) and the admittance matrix is given by Equation (13):

(13) $$\left\{\begin{array}{l} S_{21}=\frac{-2Y_{21}\sqrt{Y_{01}Y_{02}}}{\left(Y_{01}+Y_{11}\right)\left(Y_{02}+Y_{22}\right)-Y_{12}Y_{21}}\\ Insertion\;Loss=-20\log_{10}\left|S_{21}\right|=20\log_{10}\left|\frac{\left(Y_{01}+Y_{11}\right)\left(Y_{02}+Y_{22}\right)-Y_{12}Y_{21}}{2Y_{21}\sqrt{Y_{01}Y_{02}}}\right|\\ Y_{01}=Y_{02}=\frac{1}{50} \end{array}\right.$$

where *Y*_01_, *Y*_02_ denote transmission admittance.

### 2.6. Experimental Design

Using SiO_2_ as the loading material, we fabricated a series of SAW resonators with the loads in four distribution patterns. The fabrication process was as follows:

Firstly, metallic materials Al was deposited on the ST-X quartz substrate with a thickness of 2000 Å using an electron beam evaporator. Secondly, the photoresist (PR) was spin-coated, exposed and patterned for gratings. Thirdly, the Al was wet-etched and the PR was dissolved in acetone and the SAW resonators without SiO_2_ layers were fabricated. Finally, the SiO_2_ was deposited at 4 different distributions with a photomask we designed by using overlay technology, and the SAW resonators with the loads in four patterns were fabricated.

**Figure 7:**
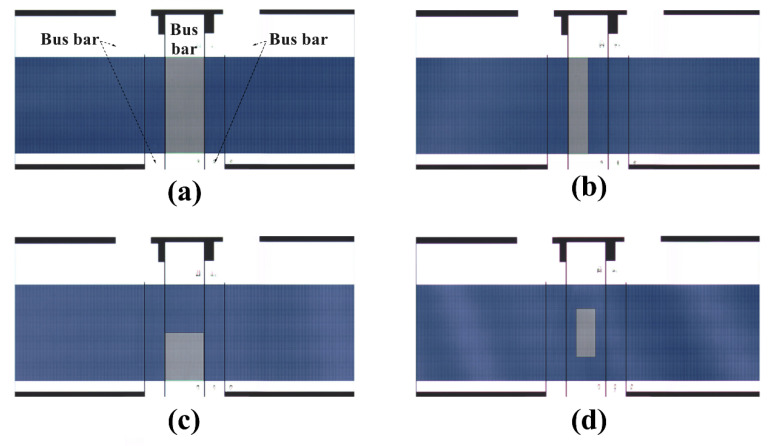
Fabricated SAW resonators with SiO_2_ loads in distribution pattern 1~4. (**a**) Load pattern-1; (**b**) Load pattern-2; (**c**) Load pattern-3; (**d**) Load pattern-4.

The photos of the SAW resonators under 4 different load patterns were taken by a microscope (LEICA DM4 M), as shown in Figure 7a–d. The information of material and structural parameters in the experiment was summarized in Table 6.

**Figure 8:**
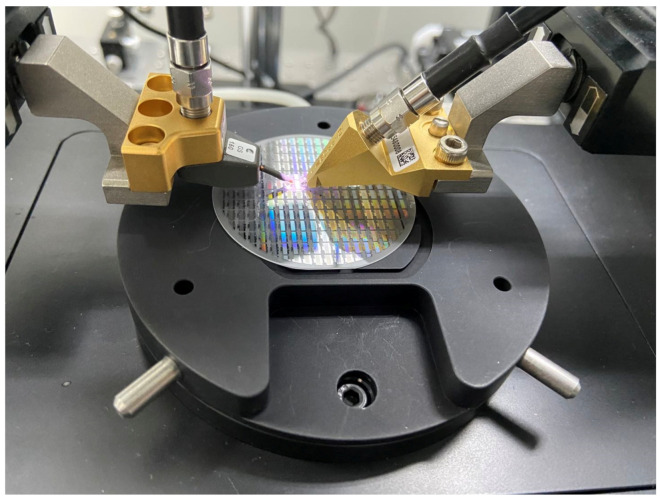
The probe device used in the measurements of the SAW resonators.

In the experiment the input port (IDT 2, IDT 3) and output port (IDT1) were connected to the VNA (Agilent E5071B Network analyzer) by using a probe device, which was shown in Figure 8.

## 3. Results and Discussion

**Figure 9:**
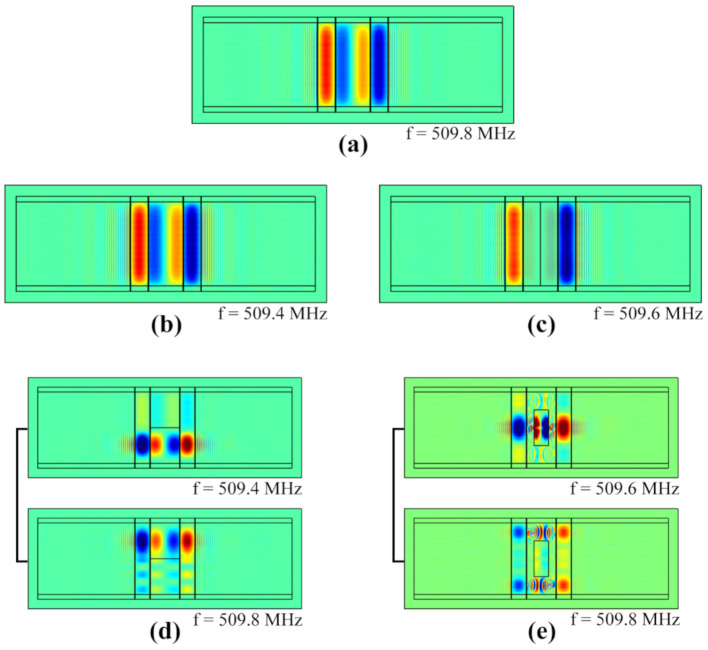
The displacement distributions of the main modes: (**a**) without mass loading; (**b**–**e**) with four different load-distribution patterns.

By solving the 2-D COM equations with FEM, we simulated the SAW sensors under four distributed patterns of mass loadings, respectively. Figure 9 showed the displacement distributions of the main modes under four load patterns.

**Figure 10:**
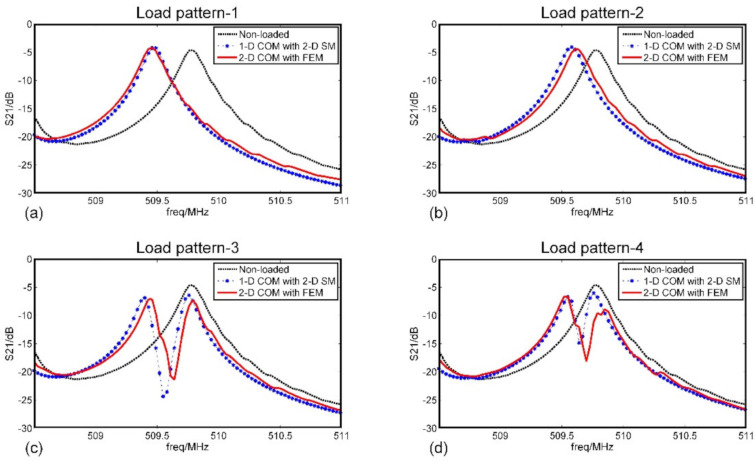
The simulated frequency response curves of the SAW sensors under four different load-distribution patterns. (**a**) Load pattern-1; (**b**) Load pattern-2; (**c**) Load pattern-3; (**d**) Load pattern-4.

By calculating the admittance matrix and the insertion loss(S21) parameters, we draw the frequency response curves of the device. As a comparison, we also obtained the frequency response curves using the 2-D SM [13], and the results were shown in Figure 10.

Of note was that the frequency response curves of load pattern 3~4 showed their center frequencies remained unchanged while the main peaks split. The reason for this phenomenon was that the SiO_2_ layer disturbed the surface acoustic wave propagation characteristics on the pattern position. It was considered that the device was composed of several channels along the aperture direction, the disturbance from SiO_2_ layer would lead to the change of the longitudinal resonance modes in those corresponding channels while it almost not has any impact on other channels [13]. The admittance of SAW device is approximately equal to the superposition of that in each channel. Therefore, the transverse distribution of the SiO_2_ layer which only appeared in pattern 3~4 affected the shape of the frequency response curves.

**Figure 11:**
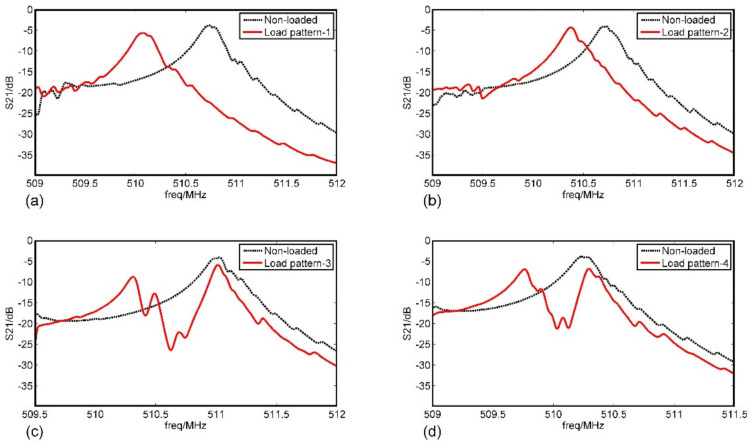
The measured frequency response curves of the load distribution pattern 1~4. (**a**) Load pattern-1; (**b**) Load pattern-2; (**c**) Load pattern-3; (**d**) Load pattern-4.

**Figure 12:**
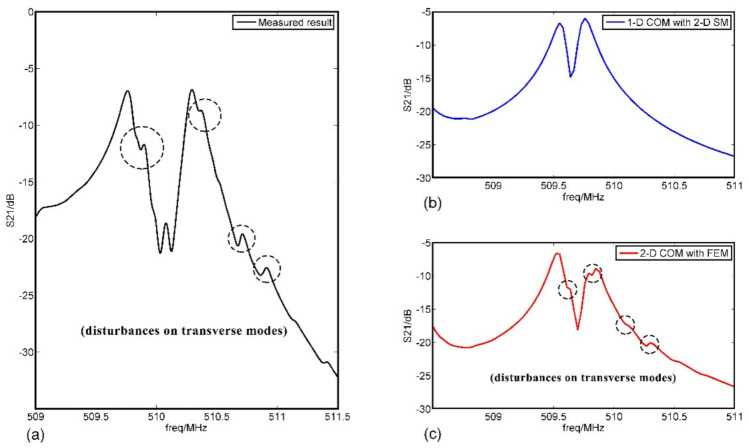
The comparison of results for load pattern-4 from measurement, 2-D SM, and 2-D COM with FEM: (**a**) the measured result; (**b**) the result of 2-D SM; (**c**) the result of 2-D COM with FEM.

The experimental results were shown in Figure 11. Of note was that the machining errors led to the frequency excursion of the fabricated SAW resonators. To achieve a better comparison and exhibit the advantage of the mothed in this paper, the response curves of load pattern-4 from measurement, 2-D SM, and 2-D COM were carried out in Figure 12. Obviously, the results obtained by using the 2-D COM equations simulated the transverse modes of the devices effectively as well as the disturbance of the mass loadings on the transverse modes (such as the parts circled in Figure 12a,c), which were more consistent with the experimental results.

## 4. Conclusions

With the wide application of SAW sensors in the field of biochemical detection, the distribution of the target analytes adhered to the sensor’s surface becomes more diverse. Therefore, it is very important to investigate the disturbance of complex distributed mass loading on the SAW propagation characteristics. Fortunately, in recent years, the finite element simulation technology has been greatly developed relying on computer technology, which provides greater help for researchers to carry out more accurate simulation work. In order to improve the accuracy of calculation and the efficiency of simulation modeling, we innovatively applied the 2-D COM theory to the simulation of SAW sensors, and used the finite element method to solve the partial differential equations. In this way, we simulated the transverse modes of SAW device and obtained the disturbance of the mass loadings on the transverse modes. The experimental results further confirmed the advantage of using the 2-D COM theory to simulate the disturbance of complex distributed mass loading on the SAW propagation characteristics.

## Figures and Tables

**Table 1:** The structural parameters of the 2-D FEM model.

Structural Parameters	Value
Period (λ)	6.074 (μm)
Aperture	150*λ
Width of busbar	10*λ
Length of Gaps between IDTs	1.25*λ
Length of Gaps between IDT and reflector	1*λ
Number of fingers in IDT1	121
Number of fingers in IDT2,3	61
Number of fingers in reflector	401

**Table 2:** The substrate material and structural parameters of the FEM model.

Substrate Material: ST-X Quartz	
Period (λ)	6.074 (μm)
Thickness of IDTs	2000 (Å)
Metallization ratio	0.5
Thickness of loads	1000 (Å)

**Table 3:** 1-D COM parameters of IDTs with and without mass loading.

1-D COM Parameters	Non-Loaded	Loaded
SAW velocity	3126 (m/s)	3124 (m/s)
Normalized reflection coefficient	0.040	0.044
Normalized excitation coefficient	2.49 × 10^−5^	2.31 × 10^−5^
Static capacitance	4.00 × 10^−11^ (F)	4.48 × 10^−11^ (F)

**Table 4:** Anisotropic parameters $\gamma_{1}$ and $\gamma_{2}$ of each domain.

Domains	$\gamma_{1}$	$\gamma_{2}$
Grating	−1.3754	−0.3988
Grating with SiO_2_ load	−1.3499	−0.6601
Busbar	−1.3366	−0.1233
Free space	−1.3928	0.1871

**Table 5:** The principle of the short-circuiting operation to calculate the admittance matrix’s elements.

Elements of Y Matrix	Short-Circuited IDTs
Y_11_	IDT1
Y_12_	IDT2, IDT3
Y_21_	IDT1
Y_22_	IDT2, IDT3

**Table 6:** The information of material and structural parameters in the experiment.

Parameters	Value
Substrate material	ST-X Quartz
Load material	SiO_2_
Grating material	Al
Thickness of gratings	2000 (Å)
Thickness of loads	1000 (Å)
